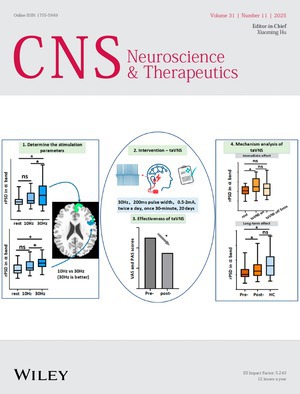# Front Cover

**DOI:** 10.1111/cns.70665

**Published:** 2025-11-21

## Abstract

The cover image is based on the article *30 Hz Transcutaneous Auricular Vagus Nerve Stimulation Alleviates Abdominal Pain by Modulating EEG Activity in the α Frequency Band of the Brain* by KAI YUAN et al., https://doi.org/10.1111/cns.70641.